# Human Herpesvirus 6A Is a Risk Factor for Multiple Sclerosis

**DOI:** 10.3389/fimmu.2022.840753

**Published:** 2022-02-10

**Authors:** Wangko Lundström, Rasmus Gustafsson

**Affiliations:** Center for Molecular Medicine, Department of Clinical Neuroscience, Karolinska Institutet, Stockholm, Sweden

**Keywords:** human herpesvirus 6A, HHV-6A, HHV-6, infection, multiple sclerosis, environment, epidemiology, risk factor

## Abstract

The role for human herpesvirus (HHV)-6A or HHV-6B in multiple sclerosis (MS) pathogenesis has been controversial. Possibly because the damage of the virus infection may occur before onset of clinical symptoms and because it has been difficult to detect active infection and separate serological responses to HHV-6A or 6B. Recent studies report that in MS patients the serological response against HHV-6A is increased whereas it is decreased against HHV-6B. This effect seems to be even more pronounced in MS patients prior to diagnosis and supports previous studies postulating a predomination for HHV-6A in MS disease and suggests that the infection is important at early stages of the disease. Furthermore, HHV-6A infection interacts with other factors suspected of modulating MS susceptibility and progression such as infection with Epstein-Barr virus (EBV) and Cytomegalovirus (CMV), tobacco smoking, HLA alleles, UV irradiation and vitamin D levels. The multifactorial nature of MS and pathophysiological role for HHV-6A in inflammation and autoimmunity are discussed.

## Introduction

### Multiple Sclerosis

Multiple Sclerosis (MS) typically presents with alternating bouts of disability and recovery. Over time this pattern often transitions into a more continuous, progressive increase in disability and neurological degeneration. Typical symptoms of MS include sensory and motoric ailments but vary greatly depending on what part of the CNS is targeted. Inflammatory events induce demyelination of neuronal axons reducing signaling capacity that induce disability (1).

The exact pathogenesis of MS is not understood, but epidemiological approaches including twin- and family studies define a complex interplay between genes and environment. For example, concordance rate among monozygotic twins is 25% and 3% for dizygotic twins (2, 3).

Several theories on the pathophysiological origin exist, but autoimmune events, whether primary triggers or secondary drivers, play a central role from disease onset and onwards.

### Genetic Risk Factors

Genome wide association studies (GWAS) have provided a fruitful approach in defining genetic associations to susceptibility (4). Prior to the GWAS era the only bona fide genetic risk factors were in the HLA region, underlining autoimmunity as a central player. The associations within the HLA region remain the strongest genetic risk factors. Furthermore, the ~100 loci associated with MS susceptibility outside HLA, almost entirely exist of genes encoding immune regulating proteins, further underlining that interplay (5).

### Environmental Risk Factors

Several environmental risk factors have been identified over the years. These include high latitude, Vitamin D deficiency and smoking. High latitude and Vitamin D deficiency are complicated to separate as discrete risk factors. Studies of migrants between low and high prevalence areas suggest the MS risk is affected by such migration (moving to a lower risk area reduces risk and vice versa), indicating the latitude effect may have more to do with sun light exposure than ethnicity (6). Vitamin D is known to regulate T-cells (7), which could explain the mechanics of the effect.

Tobacco smoking is associated with a plethora of disease risks, including inflammatory diseases. MS susceptibility increases in a dose dependent fashion by smoking. Interestingly, a gene-environment interaction seems to occur such that smoking combined with certain HLA genotypes renders larger cumulative risk than the additive risk of both factors by themselves (8).

### Herpes Viruses as Risk Factors

Human herpesvirus (HHV)-6A joins a group of herpesviruses suspected of modulating MS susceptibility and progression, most notably Epstein Barr Virus (EBV) and Cytomegalovirus (CMV) (9). HHV-6 (10) belongs to the β-herpesvirus subfamily of Herpesviridae that can establish lifelong latent infections in the host. HHV-6 isolates are classified as two distinct virus species, HHV-6A and 6B (11). Given their high degree homology it has been difficult to separate the viruses serologically. Therefore, in this review, HHV-6 will be used where no distinction has been made. Their genomes are constituted by double stranded DNA and contains hundreds of open reading frames (12). CD46 is a common cellular receptor (13) and CD134 for HHV-6B (14). T cell lines are commonly used for HHV-6 propagation. At one year of age, most individuals have acquired HHV-6 (15, 16). HHV-6A seems the predominant variant in sub-Saharan Africa (17), and HHV-6B in Europe, Japan and USA (18–20). Whereas HHV-6B is the causative agent of exanthema subitum (21), no disease has been clearly linked to HHV-6A but multiple sclerosis (MS) is a candidate (22, 23).

EBV-infection has long been suspected to increase MS risk (24) and EBV transformed B cells and formation of ectopic germinal centers have been seen in MS brains (25). Hence, EBV may be a driver of inflammation and autoimmunity after a primary injury in the CNS. EBV infection is typically asymptomatic during childhood, but generally leads to infectious mononucleosis (IM) in adolescents or adults. The chance (or risk) of avoiding childhood infection is greatest in countries with a high living standard; hence the epidemiological map of IM (implying a person was not exposed to EBV during childhood) correlates with MS prevalence. Furthermore, the risk of several autoimmune diseases (including MS) in developed countries has steadily increased over the last decades whereas the risk for infections has declined. One possible explanation is the “hygiene hypothesis” which proposes that lack of exposure to common pathogens causes increased risk of allergy and autoimmunity. This would explain the link between MS and IM without necessarily providing a direct link between the two (26).

In contrast to EBV, CMV seropositivity protect against MS. As for EBV and HHV-6A (and indeed all herpesviruses), CMV can go into latency after infection. Why CMV seropositivity diverges from EBV and HHV-6A in terms of risk effect is not understood. However, albeit asymptomatic in most infected hosts, CMV occupies large proportions of the adaptive immune system’s cellular antigen specificity (27). It has been speculated that competition between herpes viruses can modulate the immune system’s adaptation, i.e. latent CMV infection could affect the response to subsequent EBV or HHV-6A infection.

## *In Vitro* Diagnosis of HHV-6A or 6B Infection

For *in vitro* diagnostics correct sample materials and accurate methods for analyses are vital to achieve adequate data for reasonable interpretations. The best indication of active infection is isolation of the virus by inoculating susceptible cells with the biological specimen. Active infection is indicated when cells show signs of infection and/or supported viral replication. However, HHV-6 isolation is very challenging. Alternative approaches include screening plasma for cell free HHV-6A DNA by PCR. Plasma should be used (28) as latent infection may occur in blood cells. However, viral DNA in plasma may origin from latently infected and dying cells, and not necessarily from cell free virions (29, 30). Also, HHV-6 is chromosomally integrated (ciHHV-6) in roughly 1% of the population where all cells in the body contains viral DNA and viral DNA loads are typically high (31). Hence, ciHHV-6 infections may be mistaken for active infections. Also, active HHV-6 infection is highly transient and cell free viral DNA rarely detectable (32), possibly because HHV-6 spreads largely *via* cell-to-cell contact (33, 34). Therefore, reverse transcription (RT) PCR of HHV-6 mRNAs is more specific. Transcripts of the late genes U31 and U39 were found in 91-96% of samples from children with acute exanthema subitum (caused by HHV-6B), and in non during in the convalescent phase (35). Given that HHV-6A and HHV-6B are distinct species the PCR approach of choice should be able to discriminate between the two viruses (36). Antiviral IgM (37) represent an alternative measure that offers larger time windows. In primary HHV-6B infection virus specific IgM were detected five to seven days after onset of exanthema subitum and lasted for up to two months (38). However, IgM production requires a relatively strong inflammatory response and is not always detectable upon HHV-6 reactivation (39).

Following the natural course of a viral infection and inflammation, the antibody repertoire switches to IgG classes. Children seroconvert upon primary HHV-6 exposure (40), and as most people over two years old has been exposed discrete data on seroprevalence is not very informative. Instead, assessing titers of antiviral antibodies give a hint on how strong a primary infection has been; or how strongly and/or frequently the virus has reactivated. This is seen for infection with Varicella-Zoster virus (VZV) where the antiviral IgG titers increase during the convalescent phase. For CMV reactivation is associated with increased anti-CMV IgG titers (41). Therefore, the antiviral IgG titer seems to reflect the number and/or magnitude of reactivations and serve as a robust proxy indication of HHV-6 infection history.

## HHV-6 in MS

### HHV-6 in MS Brains and Increased Infection Rates During Relapse

HHV-6 has been associated to MS in numerous reports. A selection of these findings includes increased prevalence of HHV-6 DNA (42), mRNA (43) and protein expression (43–45) in MS plaques compared to in normal appearing white matter. A role for HHV-6 on MS pathogenesis in the brain is supported by increased frequencies and titers of anti-HHV-6 IgG (46, 47) and IgM (48) in cerebrospinal fluids of MS patients compared to controls; and oligoclonal band specificity against HHV-6 (49, 50). In the periphery HHV-6 mRNA and DNA is more frequent in peripheral blood mononuclear cells and serum from MS patients than in controls (51). Even though most studies show a positive association between HHV-6 and MS several conflicting reports exists where no association could be shown. A meta-analysis on the literature published between 1966 and 2009 (52) showed that 60% of the top ranked studies regarding study design, according to pre-determined criteria (53), showed significant differences between MS patients and controls in terms of HHV-6 mRNA, DNA or antibody titers. Active HHV-6 infection is significantly more frequent in MS patients during relapse than during remission (32, 54, 55) and increased titers of serum IgG antibodies against HHV-6 positively associate with relapse risk (56). It is tempting to conclude that these findings support an increased frequency of HHV-6 reactivation as a mechanism of disease activity. However, it is possible that they rather reflect a locally increased general immune activity in the CNS during relapses and that this sets of reactivation of latent viral infections, such as HHV-6, as CNS is a site of latency for HHV-6 from where the virus may reactivate (57).

### Role for HHV-6 in MS Onset, and HHV-6A Predomination

Several findings indicate that HHV-6 play a role in MS disease onset and that HHV-6A is predominant over HHV-6B. Serum IgM are detectable in the early events of an infection and increased frequency of anti-HHV-6 IgM have been detected in early stages of MS (58, 59). Marmosets challenged with HHV-6A but not those challenged with HHV-6B gave clinical MS like symptoms and lesions seen by MRI (60). This suggests a role for HHV-6 in disease onset and/or periods of active MS disease and that HHV-6A is predominant over HHV-6B. In humans, this is further supported by that HHV-6A is more neurotropic than HHV-6B and increased cellular immune response against HHV-6A, but not against HHV-6B, has been reported in MS patients compared to controls (22), and increased frequencies of HHV-6A and 6B coinfection in MS patients compared to controls (36). Also, MS serum exhibit increased seroreactivity against HHV-6A compared to HHV-6B infected cells (47) and increased detection of HHV-6A DNA in MS serum and cerebrospinal fluids compared to HHV-6B (61) has been reported.

In our lab we investigated this further in a large clinical material with well characterized MS patients (n=8,742) and closely matched controls (n=7,215) (23) and found that in MS patients the antibody response against HHV-6A was increased (OR=1.55; p=9*10^-22^) and decreased against HHV-6B (OR=0.74; p=6*10^-11^), compared to controls. This effect was even more pronounced in samples drawn from MS patients prior to diagnosis, in median 8.3 years (n=478) and closely matched controls (n=476), where people who developed MS later in life exhibited increased anti-HHV-6A antibody levels (OR=2.22; p=2*10^-5^) compared to controls. No difference was seen for HHV-6B. The serological measurements were performed with a novel bead-based multiplex serology assay that can measure antibodies against the immediate early proteins IE1A (HHV-6A) and IE1B (HHV-6B) encoded by the open reading frame (ORF) U90-U89, the most divergent between the viral species with 62% homology. To conclude, in MS HHV-6 seems important in early stages of the disease, to have a direct effect on the CNS and HHV-6A seems more prominent than HHV-6B. Even though most studies show a positive association the concept is still controversial. Therefore, additional carefully performed studies that can distinguish between HHV-6A and HHV-6B are needed.

### Genetic and Environmental Risk Factors for MS, and HHV-6A Infection

To investigate how history of infection (antiviral antibodies) for HHV-6A and HHV-6B correlates with host genetics the serologically characterized MS and control cohort described above were genotyped (23). Most associated single nucleotide polymorphisms (SNP) were found within the HLA region. Interestingly, while 191 SNP were associated with anti-HHV-6A antibody levels only two SNPs were associated with anti-HHV-6B levels. To speculate, as HHV-6 is ubiquitous (15) and as HHV-6B is more common than HHV-6A (18–20) it is possible that HHV-6B has coevolved with the human species to a larger extent than HHV-6A. A similar pattern is seen for CMV, also reaching 100% seroprevalence, and where GWAS data could not be associated to serology (62).

For MS, the major risk HLA haplotype DRB1*15:01 (63) is not associated with anti-HHV-6A levels (23), indicating the serological response could not explain this previously known risk gene. However, presence of DRB1*15:01 in combination with absence of the protective allele HLA-A*02:01 aggravated the MS risk in people with high anti-HHV-6A antibody levels (23). Analogous for EBV, MS patients with DRB1*15:01 and without A*02 had lower serum viral DNA loads compared to those without DRB1*15:01 and with A*02 (24). Other known MS risk factors such as smoking, low ultraviolet radiation and low vitamin D levels all aggravated the MS risk in people with high anti-HHV-6A antibody levels (64). Similar interaction analyses were performed between anti-HHV-6A and anti-EBV (risk factor) and anti-CMV (protective) antibody levels in plasma from people who developed MS 8 years after the sampling date. High anti-HHV-6A levels interacted with high anti-EBV levels and aggravated the MS risk (65). When adding CMV to the model people with high levels of anti-HHV-6A and anti-EBV antibodies, and low levels of anti-CMV antibodies had 15 times higher risk of developing MS later in life compared to people with low levels of IE1A and EBV antibodies and high levels of CMV (66).

**Figure 1:**
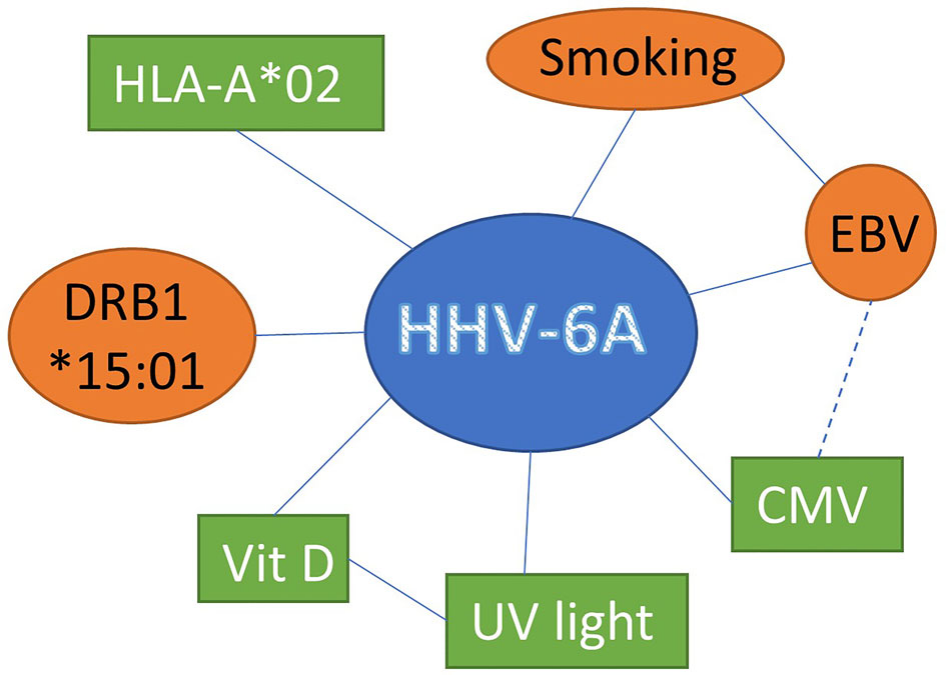
Interaction between HHV-6A and other factors suspected of modulating MS disease susceptibility susceptibility and progression increasing (red circles) or decreasing (green boxes) the risk for MS development.

Together these data suggests that HHV-6A represents a risk factor by itself and that interaction with other factors suspected of modulating MS disease further impacts the risk for developing MS (**Figure 1**).

### HHV-6A in MS Pathogenesis

In MS, autoreactive T cells are thought to target the myelin sheath. Antigen presenting cells (APC) such as dendritic cells (DC) are the primary conductors of T cell regulation. DCs mature upon exposure of pathogen-associated molecular patterns (PAMPs) and thereafter can activate T cells. As PAMPs are not present in self-tissue they likely need to be present for breakage of tolerance and autoimmunity. A model has been proposed where APCs need a danger signal in combination with the antigen to elicit an immune response against the antigen (67). An example is the animal model for MS, experimental autoimmune encephalopathy (EAE). Here, the adjuvant provides the immune system with a danger signal to triggering an immune response and the myelin proteins steers the response against myelin. EAE has striking similarities with MS with infiltration of lymphocytes in the CNS and demyelination, as well as clinical symptoms e.g. paralysis (68).

Several mechanisms for HHV-6-induced autoimmunity and myelin breakdown have been suggested. These include molecular mimicry and host cell incorporation in the virion. The molecular mimicry model proposes that the virus encodes for a protein with similar motifs to host cell proteins, and that an immune response directed against this “virus encoded host-like protein” is cross-reactive and also target the host protein that is mimicked. For HHV-6 and MS, molecular mimicry has been shown with cross-reactivity between a peptide encoded by the viral gene U24 and myelin basic protein (MBP) (69). The host cell protein incorporation hypothesis is based on the well-documented notion that the envelop of enveloped viruses origin from the host cell, and therefore, that host cell proteins are present in the viral envelop. When an APC engulfs a virion containing host cell proteins an immune response is triggered by the PAMPs of viral motifs and directed against the host cell proteins contained in the virion (70). Both HHV-6A and HHV-6B have the capacity to infect oligodendrocytes, which is a pre-requisite. But the model suffers from studies where release of cell free virions is not commonly seen during HHV-6 infection of oligodendrocytes (71–73).

Both the molecular mimicry and the host cell incorporation model relies on the idea that HHV-6 virion can trigger the T cell stimulatory capacity of APC. However, we and others have shown that inoculation of DC with both HHV-6A and HHV-6B instead hamper the capacity of DC to activate T cells, both in absence and presence of viral replication, and for both allogenic and autologous T cells (74–79). Hence, HHV-6 induced pathogenesis in MS does not seem to occur through neither of these models.

HHV-6 induces syncytia but also gives semi-lytic infection with dying cells and cell debris. DC exposure to HHV-6A result in cell death and release of high mobility group box 1 (HMGB1) protein (80). This may occur also in the CNS as HHV-6A can induce vigorous apoptosis of a human oligodendrocytes (81). HMGB1 can activate DC in the autoimmune disease systemic lupus erythematosus (82). In MS, HMGB1 has been proposed as an important driver (83) with HMGB1 expression in MS lesions (84) and elevated serum levels of HMGB1 in treatment naïve MS patients, compared to those receiving disease-modifying treatment (85).

Hence, we propose a model for HHV-6A induced pathogenesis where HHV-6A infection and/or reactivation (86) in the CNS gives a primary injury. This in turn gives inflammatory events, possibly driven by HMGB1, with upregulation of metalloproteinase and influx of leukocytes from the periphery which can cause pathological inflammation and subsequent plaque formation. The inflammation is aggravated by EBV infection and tobacco smoking; and hampered if the immune system is occupied by CMV and/or regulated when vitamin D levels are sufficient.

## Conclusions

Together, the studies compiled in this review suggest a role for HHV-6A in MS, particularly early in the disease course and/or at MS onset. This is based on findings of increased anti-HHV-6A antibody levels in people who developed MS later in life, compared to people who do not. HHV-6A infection interacts with other risk factors such as carriage of HLA-DRB1*15:01, tobacco smoking, low UV irradiation, low vitamin D levels and EBV infection; and with protective factors such carriage of the protective haplotype HLA-A*02:01 and CMV infection. This shows the multifactorial nature of MS and suggests that accumulated burden of risk factors increases the risk for acquiring the disease. We propose a pathophysiological role for HHV-6A in induction of MS where an infection of the CNS leads to a primary injury and that this in turn leads to inflammatory events and autoimmunity.

## Author Contributions

RG conceived this review article. WL and RG contributed to design of the review, wrote the first draft of the manuscript, contributed to manuscript revision, read, and approved the submitted version.

## Conflict of Interest

The authors declare that the research was conducted in the absence of any commercial or financial relationships that could be construed as a potential conflict of interest.

## Publisher’s Note

All claims expressed in this article are solely those of the authors and do not necessarily represent those of their affiliated organizations, or those of the publisher, the editors and the reviewers. Any product that may be evaluated in this article, or claim that may be made by its manufacturer, is not guaranteed or endorsed by the publisher.
